# 004 Systemic autoinflammatory diseases: a multicenter cohort study

**DOI:** 10.1093/rheumatology/keac495.004

**Published:** 2022-10-07

**Authors:** Ayşe Tanatar, Özlem Akgün, Şengül Çağlayan, Semanur Özdel, Gülçin Otar Yener, Mustafa Çakan, Kübra Öztürk, Hafize Emine Sönmez, Betül Sözeri, Nuray Aktay Ayaz

**Affiliations:** 1 Istanbul University, Istanbul Faculty of Medicine, Department of Pediatric Rheumatology, Istanbul, Turkey; 2 University of Health Sciences, Ümraniye Research and Training Hospital, Department of Pediatric Rheumatology, Istanbul, Turkey; 3 University of Health Sciences, Dr. Sami Ulus Maternity and Child Health and Diseases Research and Training Hospital, Department of Pediatric Rheumatology; 4 Şanlıurfa Research and Training Hospital, Department of Pediatric Rheumatology; 5 Istanbul Medeniyet University, Göztepe Prof. Dr. Süleyman Yalçın City Hospital, Department of Pediatric Rheumatology; 6 Kocaeli University Faculty of Medicine, Department of Pediatric Rheumatology

## Abstract

**Background:**

Systemic autoinflammatory diseases (SAIDs) are a group of hereditary diseases characterized by inflammatory attacks accompanied by clinical findings such as fever, skin rash, abdominal pain, and musculoskeletal symptoms and with high acute phase response.

**Objectives:**

This study sought to define the characteristics, to report 24-month follow-up of children with SAIDs and to compare the performances of both the previously validated classification criteria Federici and new Eurofever/PRINTO classification criteria in a multicentre cohort under real-life conditions.

**Method:**

The medical charts of 60 children diagnosed with SAIDs (CAPS, TRAPS and HIDS) who were followed up regularly every 1–3 months between 2010 and 2021 in 6 pediatric rheumatology centers in Turkey were retrospectively analyzed. Demographic data, clinical findings, laboratory results and treatments were recorded. All patients were analyzed by NGS panel containing 16 genes.

**Results:**

A total of 60 patients with SAID were evaluated. Among them 30 (50%) were male and 30 (50%) were female. The median age at disease onset and diagnosis were 22 (0–193) months and 72 (2–213) months, respectively. The current age was median 10.3 (1.7–20.4) years. All patients were follow-up with a median 31 (6–97) months. Among 60 patients, 22 were diagnosed with CAPS, 8 with TRAPS and 30 with HIDS. The frequency of clinical symptoms was 95%, arthralgia 63.3%, gastrointestinal complaints 58.3%, urticarial rash 56.7%, myalgia 48.3%, lymphadenopathy 33.3%, respectively.

Urticarial rash (86.4%), cold induced rash (45.5%) and arthritis (50%) were significantly higher in CAPS patients compared with other two groups (*p*< 0.05). Lymphadenopathy (46.7%) was more common in HIDS patients (*p* = 0.03).

When we applied the diagnostic/classification criteria to our cohort, 71.7% met the Federici criteria, 53.3% fulfilled the Eurofever/PRINTO classification criteria. When performance of two diagnostic criteria (Federici and Eurofever/PRINTO criteria) was compared, the proportion of patients fulfilling the Eurofever/PRINTO criteria higher in TRAPS patients compared with other groups (*p* = 0.008).

Statistically significant decreases were found in AIDAI scores, CRP, ESR levels from the 1st month and in the leucocyte counts from the 6th month of the treatment in CAPS patients (*p* = <0.001, *p* = 0.035, *p* = 0.03 and *p* = 0.006, respectively). In addition, AIDAI scores were correlated with CRP values (*p* = 0.047).

In HIDS patients, there was a statistically significant decrease in AIDAI scores and ESR levels from the 1st month, CRP levels from the 3rd month, and leucocyte counts from the 12th month of treatment (*p* = <0.001, *p* = 0.003, *p* = <0.001 and *p* = 0.034, respectively). In addition, ESR values were correlated with CRP (*p* = 0.006). In TRAPS patients, on the other hand, there was a statistically significant decrease in AIDAI scores from the 1st month of treatment (*p* = 0.018).

At the last visit, 93.3% of the patients were receiving anti-IL-1, 5% anti-IL-6, and 1.7% were receiving colchicine. Ninety percent of the patients were in complete remission.

**Conclusion:**

We showed that the diagnostic/classification criteria may not always be sufficient to make the diagnosis, and there is still low agreement between clinical diagnoses and molecular analyzes. Anti-IL-1 treatments have been found to be quite effective in the treatment from the early period.

